# Better experiences with quality of care predict well-being of patients with chronic obstructive pulmonary disease in the Netherlands

**DOI:** 10.5334/ijic.1587

**Published:** 2015-06-22

**Authors:** Jane Murray Cramm, Shahab Jolani, Stef van Buuren, Anna Petra Nieboer

**Affiliations:** Department of Health Policy and Management (iBMG), Erasmus University Rotterdam, Rotterdam, The Netherlands; Department of Methodology and Statistics, Utrecht University, Utrecht, The Netherlands; Department of Methodology and Statistics, Utrecht University, Utrecht, The Netherlands; Department of Life Style, TNO, Leiden, The Netherlands; Department of Health Policy and Management (iBMG), Erasmus University Rotterdam, Rotterdam, The Netherlands

**Keywords:** chronic obstructive pulmonary disease, disease management, health behaviour, quality of care, well-being

## Abstract

**Objective:**

This study was conducted to (1) identify improvements in care quality and well-being of patients with chronic obstructive pulmonary disease in the Netherlands and (2) investigate the longitudinal relationship between these factors.

**Methods:**

This longitudinal study was conducted among patients diagnosed with chronic obstructive pulmonary disease enrolled in the Kennemer Lucht care programme in the Netherlands. Biomarker data (lung capacity) were collected at patients’ health care practices in 2012. Complete case analysis was conducted, and the multiple imputation technique allowed us to report pooled results from imputed datasets.

**Results:**

Surveys were filled out by 548/1303 (42%) patients at T0 (2012) and 569/996 (57%) remaining participants at T1. Quality of care improved significantly (*p* < 0.05). Analyses adjusted for well-being at T0, age, educational level, marital status, gender, lung function and health behaviours showed that patients’ assessments of the quality of chronic care delivery at T0 (*p* < 0.01) and changes therein (*p* < 0.001) predicted patients’ well-being at T1.

**Conclusion:**

These results clearly show that the quality of care and changes therein are important for the well-being of patients with chronic obstructive pulmonary disease in the primary care setting.

**Practice implications:**

To improve quality of care for chronically ill patients, multicomponent interventions may be needed.

## Introduction

Populations are ageing worldwide and the prevalence of chronic diseases is increasing rapidly [1]. Chronic obstructive pulmonary disease is the leading cause of death from lung disease worldwide [2]. It is characterized by chronic obstruction of lung airflow, which interferes with normal breathing and is not fully reversible [3]. In the Netherlands, the prevalence of chronic obstructive pulmonary disease among older adults (aged ≥ 55 years) is about 12% [4]. Due to patients’ substantial contributions to the volumes of emergency department visits and hospitalizations, chronic obstructive pulmonary disease is considered a costly disease [5]. It also negatively affects patients’ well-being, imposing a burden on daily life that extends beyond their physical/health conditions [6, 7].

The medical community has traditionally focused on acute care and short-term goals that emphasize the management of acute exacerbations and complications and the reduction of recovery time; high-quality chronic care delivery is typically lacking within this system [8–10]. Patients with chronic obstructive pulmonary disease often do not receive optimal care [11], and the disease is under-diagnosed and under-treated [5, 12, 13]. In the Netherlands, most patients with mild to moderate chronic obstructive pulmonary disease are treated in primary care practices [14]. Treatment takes place according to guidelines for the regular monitoring of symptoms and airflow obstruction in the primary care setting with the goals of guiding the modification of treatment and enabling the early detection of complications [14, 15]. Patients with chronic obstructive pulmonary disease should be monitored regularly to achieve these goals and to delay disease progression and alleviate its manifestations [16]. Care should also be holistic and patient-centred, with shared responsibility focused on the needs of individual patients [8–10, 17–20]. Research has shown that higher quality chronic care delivery results in fewer hospital admissions and emergency department visits among patients with chronic obstructive pulmonary disease [21]. Furthermore, the literature strongly suggests that change may be achieved only through multicomponent interventions at the patient, professional and organizational levels [22–26].

The extent to which primary care practices aiming to improve the quality of chronic care delivery successfully enhance the experiences of patients with chronic obstructive pulmonary disease has not been studied thoroughly. We assume that the rationale underlying quality improvement programmes in primary care settings (i.e. evidence-based, structured care focused on patient activation) is legitimate and favours better outcomes, resulting in better patient assessments of chronic care. Furthermore, as high-quality chronic care delivery calls for a comprehensive approach to support patients with chronic obstructive pulmonary disease over time and take responsibility for their well-being, we expect that quality improvement protects patients’ well-being. Holistic, patient-centred programmes offering self-management support services have improved patient outcomes [20]. We identified a cross-sectional relationship between the quality of chronic care delivery and the well-being of patients with chronic obstructive pulmonary disease [27], but the longitudinal relationship between these factors in the primary care setting remains unknown. Thus, this study aimed to (1) identify improvements in patients’ assessments of the quality of care delivery and their well-being over time in the Netherlands, and (2) investigate the longitudinal relationship between the quality of chronic care delivery and well-being of patients with chronic obstructive pulmonary disease while controlling for patients’ baseline well-being, (changes in) health behaviours (physical activity and smoking) and background characteristics (age, gender, marital status, educational level and lung function).

## Methods

### Setting

This longitudinal study was conducted in April/May 2012 (T0) and April/May 2013 (T1) among patients recently enrolled in a newly implemented chronic obstructive pulmonary disease care programme called “Kennemer Lucht”. Patients received care at one of 46 participating primary health care practices in the Noord-Kennemerland region of the Netherlands. The disease management programme began in March 2012 and involved multicomponent interventions at the patient, professional and organizational levels to improve the quality of care for patients with chronic obstructive pulmonary disease (see Box 1 for a full overview of the 35 implemented interventions). This box shows that this disease management programme incorporated all six interrelated components of the chronic care model: (1) self-management support, (2) delivery system design, (3) decision support, (4) clinical information systems, (5) healthcare organization and (6) community linkages [8–10]. Examples of implemented interventions are: patient education, motivational interviewing, lifestyle (healthy diet, drinking, smoking and exercise) advice, medical treatment according to clinical guidelines and the use of flowcharts, regular follow up of patients and regular consultation/coordination with hospital care (e.g. concerning medicine). Earlier research showed that a constellation of interventions is needed and that a disease management programme is deemed to be based on the chronic care model if their constellation of interventions attempted to make changes can be mapped to at least four components of the chronic care model [28, 29] and those implementing interventions within all six dimensions or the chronic care model are considered as high-quality of care [30].

### Participants and study design

The Kennemer Lucht programme included all patients diagnosed with chronic obstructive pulmonary disease receiving primary care. These patients had recently enrolled in the newly implemented chronic obstructive pulmonary disease-care programme. No additional inclusion criterion was applied. At T0 and T1, patients received questionnaires at home *via* mail. A few weeks later, reminder notices were sent to non-respondents. Another few weeks later, second reminder notices with duplicates of the questionnaire were sent. Biomarker data (lung capacity measured with spirometry) were collected at the health care practices in 2012. The ethics committee of the Erasmus University Medical Center of Rotterdam approved this study in April 2012 (MEC-2012-143).

### Survey measures

Well-being was measured with the 15-item version of the Social Production Function Instrument for the Level of Well-being (SPF-IL) [31]. This scale measures levels of physical (comfort, stimulation) and social (behavioural confirmation, affection, status) well-being. Examples of questions are: “In the past few months, have you felt physically comfortable?” (comfort), “Do you really enjoy your activities?” (stimulation), “Do you feel useful to others?” (behavioural confirmation), “Do people pay attention to you?” (affection) and “Are you known for the things you have accomplished?” (status). Scores range from 1 to 4, with higher scores representing greater well-being. Cronbach's alpha values for the SPF-IL at T0 and T1 were 0.86 and 0.85, respectively, indicating good reliability.

Patients’ assessments of care were measured with the 20-item Patient Assessment of Chronic Illness Care (PACIC) questionnaire, which uses a five-point response scale ranging from “almost never” to “almost always” [32]. Examples of items are: “When I received care for my chronic illness over the past 6 months, I was…” “…asked for my ideas when we made a treatment plan”, “…satisfied that my care was well organized”, “…asked how my chronic illness affects my life” and “…asked how my visits with other doctors were going”. Scores range from 1 to 5, with higher scores representing a higher quality of chronic care delivery. The Cronbach's alpha value for the PACIC was 0.94 at T0 and T1, indicating excellent reliability.

Physical activity was assessed by asking respondents how many days per week they were physically active (e.g. sport activities, exercise, housecleaning, work in the garden) for at least 30 minutes. This question was taken from the Short QUestionnaire to ASsess Health-enhancing physical activity (SQUASH) instrument, which was developed in the Netherlands and has been validated using an accelerometer [33]. We dichotomized the physical activity scale according to the Dutch Standard for Healthy Physical Activity as 1 (at least 5 days per week) or 0 (less than five days per week) [34].

Education was classified using six categories ranging from 1 [no school or some primary education (≤7 years)] to 6 [completion of a university degree (≥18 years)]. We dichotomized this item as 1 [low educational level (no school/some primary education or lower technical/vocational education)] or 0 (more than lower technical/vocational education). We further asked respondents to report their marital status, gender and age. Self-reported current smoking was assessed with a yes/no question.

### Lung function

Spirometry was used to measure lung function, specifically the amount (volume) and/or speed (flow) of air that patients could inhale and exhale, in the health care practices. The flow electronic volume percentage, calculated as forced vital capacity/forced expiratory volume in 1 second, was used to identify lung problems. The Global Initiative for Chronic Obstructive Lung Disease (GOLD) criteria were used to classify the severity of chronic obstructive pulmonary disease (normal lung function and GOLD stages 1–4).

### Statistical analyses

We used descriptive statistics to describe the study population. Two-tailed, paired *t*-tests or chi-squared tests were used to investigate improvements in patients’ health behaviours (smoking and physical exercise), the quality of chronic care and patients’ well-being over time (difference between T0 and T1). To account for the nested structure of our study population [patients (level 1) nested in health care practices (level 2)], we employed a multilevel random-effects model to investigate the predictive roles of (changes in) health behaviour and quality of chronic care in patients’ well-being while controlling for patients’ well-being at T0, age, gender, educational level and marital status. Two-sided *p-*values ≤ 0.05 were considered to be significant. Analyses were performed using SPSS software (version 19; IBM).

Because data were missing from a large proportion (59%, 221/372) of patients, we employed the multiple imputation technique [35, 36] and report pooled results from imputed datasets (*n* = 372 each) in addition to those from the complete case analysis. Missing values were imputed using the MICE software package [37] in *R 3.2.1*. Predictive mean matching was used as an imputation model to ensure that imputed values preserved the actual range of each variable. A multilevel imputation method (i.e. a random-intercept imputation model) was applied to account for the nested structure of the data.

## Results

At T0, 548 of 1303 patients with chronic obstructive pulmonary disease filled out surveys (42% response rate). At T1, 569 of 996 patients still participating in the chronic obstructive pulmonary disease care programme filled out surveys (57% response rate). A total of 372 respondents filled in questionnaires at both T0 and T1.

Table 1 displays the baseline characteristics of patients with chronic obstructive pulmonary disease. Of the 548 respondents, 46% were female, 34% had low educational levels and 34% were single. The mean age was 68.38 ± 10.27 (range, 38–91) years. The mean well-being score was 2.77 ± 0.48 (range, 1–4) and mean assessment of quality of chronic care was 2.76 ± 0.91 (range, 1–5). The majority (60%) of respondents reported being physically active for 30 minutes on at least 5 days per week and 31% of respondents were current smokers.

Pooled results of imputed data for the entire study population (*n* = 372) showed no change in the percentages of current smokers and physically active patients between T0 and T1. Similarly, no difference in mean well-being (2.78 at T0 *vs.* 2.75 at T1) was observed. However, the quality of chronic care delivery improved significantly over time (2.72 at T0 *vs.* 2.79 at T1; *p* < 0.05). Complete case analyses yielded similar findings, with significant improvement only in the quality of care delivery.

Pooled results of imputed data (*n* = 372) subjected to multilevel analyses adjusted for baseline characteristics and GOLD classification showed that physical activity at T0 (*p* < 0.01), changes therein (*p* < 0.05), patients’ assessments of the quality of chronic care at T0 (*p* < 0.01) and changes in these assessments (*p* < 0.001) predicted patients’ well-being at T1 (Table 2). Physically active status at T0 was related to better well-being at T1 (*B* = 0.12), and one incremental increase in physical activity between T0 and T1 improved well-being (*B* = 0.09), assuming that all other factors in the model remained constant. Higher quality chronic care at T0 was also related to better well-being at T1 (*B* = 0.06), and one incremental increase in quality further improved well-being (*B* = 0.14), assuming that all other factors remained constant. Complete case analyses yielded similar findings, identifying the same significant predictors of well-being at T1.

## Discussion

This study showed that multicomponent interventions based on the chronic care model improved patients’ experiences with the quality of chronic care delivery in the primary care setting over time. Moreover, these improvements in care quality predicted the well-being of patients with chronic obstructive pulmonary disease. Our previous research among chronically ill patients in general has also documented improved outcomes after the implementation of such interventions, in terms of quality of care delivery perceived by disease management professionals [38, 39] and chronically ill patients [40], as well as patients’ health behaviours [41].

The percentages of patient participants meeting the Dutch standard for healthy physical activity at T0 (60%) were comparable to the percentage in the general adult (18+ years) Dutch population (58% in 2011) [42]. The proportion of current smokers at T0 (31%) was slightly higher than the mean prevalence of smoking in the general Dutch population (25.6% in 2011) [43]. Mean level of well-being at T0 (2.78) was comparable to the average level of well-being found among older persons who are chronically ill (mean 2.76) and who had recently been hospitalized (2.78) [44]. The percentage of patients with lower educational levels, however, was lower compared to chronic obstructive pulmonary disease patients enrolled in other Dutch disease management programmes (35% *vs.* 50%) [25].

This study has several limitations. First, the lack of a control group prevented us from determining whether the observed absence of changes in smoking, physical exercise and well-being in chronic obstructive pulmonary disease care programme participants differed from the characteristics of patients with chronic obstructive pulmonary disease receiving care as usual, whose well-being may have deteriorated over time. In addition, disease severity may have affected our study findings, which is only partly captured by lung function. Second, the 1-year study period was not sufficient to detect changes in health behaviours and well-being in our participants; we expect these factors to improve over a longer period of time with improved experiences with the quality of care delivery, as suggested by the finding that (changes in) this quality predicted patients’ well-being. In a study of the effectiveness of disease management programmes in Dutch primary care settings, we observed that patients’ physical quality of life decreased over a 1-year period, but noted improvement over a 2-year period [45]. Furthermore, although the quality of chronic care delivery improved significantly over time, this improvement was only small and may not be clinically relevant. Further research is thus necessary to investigate the long-term effects of chronic obstructive pulmonary disease care programmes using longer (e.g. 2-year) study periods as well as to identify the minimal clinically important difference in quality of chronic illness care. Third, investigations of the effectiveness of similar programmes for patients with other chronic conditions and/or comorbidities in the Netherlands and other countries are also needed to confirm the generalizability of our study findings. Fourth, non-response bias may have affected our findings. The percentage of patients with lower educational levels, for example, was lower compared to chronic obstructive pulmonary disease patients enrolled in other Dutch disease management programmes [25]. Fifth, we analysed only patients’ self-reported perceptions and did not investigate objective health outcomes, although we controlled for patients’ lung function. Finally, it is possible that improvements in quality of care observed in the case study may especially be beneficial to already activated chronically ill patients, whereas non-active patients may benefit less from disease management programmes based on the chronic care model.

## Conclusion

The implementation of a chronic obstructive pulmonary disease care programme consisting of multicomponent interventions at the patient, professional and organizational levels in the primary care setting has the potential to improve patients’ experiences with the quality of chronic care delivery over time. Furthermore, the results of this study clearly show that the quality of care delivery and changes therein are important for the well-being of patients with chronic obstructive pulmonary disease in this setting.

## Practice implications

The findings of this study emphasize the need for implementation of a constellation of interventions for chronic obstructive pulmonary disease patients in primary care. Such efforts are of particular importance in the current context of ageing populations and increased prevalence of (multiple) chronic diseases treated in primary care settings. The use of multicomponent interventions in these settings at the patient (e.g. patient education, counselling on treatment compliance and coping strategies), professional (e.g. working according to evidence-based guidelines) and organizational (e.g. systematic following of patients, regular consultation/coordination with hospital care) levels is expected to be beneficial. Policy-makers should realize that simple interventions or incremental quality improvement may not be sufficient to improve the well-being of chronic obstructive pulmonary disease patients in the primary care setting. Practices should be made aware of what is needed to improve outcomes for chronically ill patients and those aiming to improve the quality of care for this population should be provided with the financial means to do so (e.g. through bundled payments).

## Figures and Tables

**Box 1.**
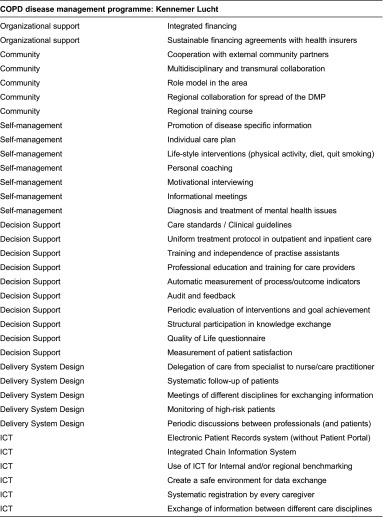
Interventions implemented in the disease management programme mapped to the chronic care model

**Table 1.**
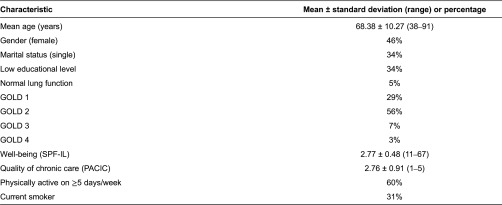
Baseline characteristics of patients participating in the “Kennemer Lucht” COPD care programme

COPD, chronic obstructive pulmonary disease; GOLD, Global Initiative for Chronic Obstructive Lung Disease; SPF-IL, Social Production Function Instrument for the Level of Well-being; PACIC, Patient Assessment of Chronic Illness Care

**Table 2.**
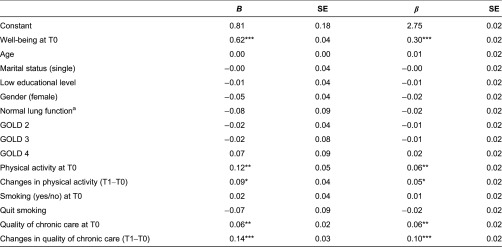
Predictors of well-being at T1 (2013), as assessed by multilevel random-intercepts regression analyses (*n* = 372)

^a^GOLD 1 served as the reference group. SE = standard error; T0 = baseline (2012); GOLD = Global Initiative for Chronic Obstructive Lung Disease; T1 = follow-up (2013)

*p ≤ 0.05; **p ≤ 0.01; ***p ≤ 0.001 (two-tailed). These findings are based on pooled results of imputed data. Complete case analyses identified the same significant predictors of well-being at T1
